# A randomized pilot study of electro-acupuncture treatment for hysterosalpingography pain relief and related anxiety

**DOI:** 10.4274/tjod.galenos.2020.66592

**Published:** 2020-12-10

**Authors:** Zeyneb Bakacak, Adnan Demirel, Murat Bakacak, Aykut Urfalıoğlu, Aslı Yaylalı, Ömer Faruk Boran, Mustafa Kaplanoğlu, Hakan Kıran, Mehtap Gizir

**Affiliations:** 1 Private Clinic, Kahramanmaraş, Turkey; 2 Bolu Abant İzzet Baysal University Faculty of Medicine, Department of Physical Therapy and Rehabilitation, Bolu, Turkey; 3 Kahramanmaraş Sütçü İmam University Faculty of Medicine, Department of Obstetrics and Gynecology, Kahramanmaraş, Turkey; 4 Kahramanmaraş Sütçü İmam University Faculty of Medicine, Department of Anesthesiology and Reanimation, Kahramanmaraş, Turkey; 5 Kahramanmaraş Sütçü İmam University Faculty of Medicine, Department of Histology and Embriyology, Kahramanmaraş, Turkey; 6 Çukurova University Faculty of Medicine, Department of Obstetrics and Gynecology, Adana, Turkey

**Keywords:** Acupuncture, anxiety, hysterosalpingography, pain relief

## Abstract

**Objective:**

To investigate the effect of acupuncture on reducing pain and anxiety related to hysterosalpingography (HSG).

**Materials and Methods:**

A total of 107 patients planned to undergo HSG were randomly separated into 3 groups; the acupuncture group (n=36), intramuscular diclofenac sodium group (n=35), and control group (n=37). In the acupuncture group, electro-acupuncture was applied to specified points for 20 mins before the procedure. In the intramuscular diclofenac sodium group, an intramuscular injection of 75 mg diclofenac sodium was applied 30 mins before the procedure. No analgesics were administered to the patients in the control group before intervention. Pain was evaluated with a Visual Analog scale (VAS) and anxiety with the State-trait Anxiety inventory -state (STAI-S), preoperatively and at specified times postoperatively.

**Results:**

The VAS scores at 1 and 5 minutes after HSG were similar in acupuncture group and intramuscular diclofenac sodium group, and significantly lower than in the control group. At 30 mins postoperatively, there was no significant difference between the 3 groups in respect of the VAS scores. The STAI-S scores at 1 hour preoperatively were similar in all the groups (p=0.563). In the comparisons of the STAI-S values at preoperative 5 mins, following acupuncture in acupuncture group and the diclofenac injection in intramuscular diclofenac sodium group, and at postoperative 30 mins, the acupuncture group values were determined to be statistically significantly lower than those of the other groups (p<0.001, p<0.001).

**Conclusion:**

Acupuncture has similar effects on the reduction of pain as other analgesics and reduces anxiety. It can therefore be used in HSG in suitable clinics.

**PRECIS:** Acupuncture has similar effects on the reduction of pain as other analgesics and reduces anxiety.

## Introduction

Infertility affects approximately 15% of the general population worldwide and 40-50% is attributed to female infertility. It is caused by many factors, and fallopian tube abnormalities cause about 30-35% of cases of female infertility^(1)^. Hysterosalpingography (HSG) is a relaible and cost-effective method used in the determination of both tubal pathologies and tubal patency, and uterine and peritoneal pathologies in the investigations into the etiology of infertility^(2)^. Although HSG is a procedure that does not require cervical dilatation or general anaesthesia, the procedure can be painful. Up to 72% of patients complain of pain from the procedure^(3)^.

The mechanisms causing pain may include cervical instrumentation, and irritation of contrast medium in uterine cavity distension, and peritoneal irritation of contrast medium that has spread to the abdomen. In addition, taking the uterus into traction by holding the cervix with a tenaculum increases local prostaglandin synthesis and can lead to uterine contractions and pain^(4)^. Patients think that this procedure will be painful and are reluctant to have the test, which also causes anxiety. Many different randomised controlled studies have investigated many different agents and different application methods to reduce pain during HSG^(5,6,7,8)^, and there are reviews in literature that have evaluated these studies^(9,10,11)^. However, the majority of these studies have limitations such as heterogenity in the randomisation, lack of blinding, patient selection criteria, differences in drug doses and in the pain score measurements. Therefore, no optimal method has been found as yet, which can be recommended to alleviate pain during the HSG procedure.

Complementary treatments have started to be more widely used in modern medicine, and of these, acupuncture applications have shown efficacy, especially in acute pain syndromes^(12)^ and chronic pain^(13)^. Kiran et al.^(14) ^determined that acupuncture and non-steroid anti-inflammatory drugs (NSAIDs) have similar effects in patients with primary dysmenorrhea. In recent randomised controlled studies, Smith et al.^(15) ^reported that acupuncture decreased anxiety and increased quality of life in patients applied with *in vitro* fertilization.

Therefore, the aim of this study was to investigate, for the first time in literature, the effect of acupuncture in reducing pain and anxiety in patients undergoing HSG.

## Materials and Methods

Approval for the study was granted by the Clinical Research Ethics Committee of Kahramanmaraş Sütçü İmam University (decision no: 08, session: 2015/05). Patients who admitted to our Assisted Reproductive Techniques clinic with the desire to have a child and planned to undergo HSG were included in our study. Informed consent was obtained from all the patients. Age, infertility duration, gravida and smoking status of all patients participating in the study were registered. At the beginning of the study, a pilot study was conducted with 10 patients from each group and the minimum number of patients in each group with α=0.05 and 90% power was found to be 27 in the power analysis. There with, 133 patients were planned to be included in the study at first. However, the study was conducted with the remaining 107 patients after excluding the ones allergic to radiopaque substance (n=1) and NSAID (n=3), those with cervical-endometrial infection (n=5), those with previously defined NSAID drug-related side effects (n=5), those with cervical surgical anamnesis (n=1), those who were found to have acute pelvic inflammatory disease (n=3) and those who did not want to participate in the study. The remainig 107 subjects were divided into three groups after computer-generated randomization; the acupuncture (ACU) group (n=36), intramuscular diclofenac sodium (IMDS) group (n=35), and the control group (n=37).

For the patients in the ACU group, taking anxiety into consideration, the points selected and identified to decrease the pain and stress that can develop during the taking of HSG were those which are most widely used in the treatment of dysmenorrhea and anxiety. All the ACU procedures were applied by the same physician (A.D.) who has experience on ACU procedures for more than ten years. The H7, Du-20, Liv-3, P-6, HT-7, PC-6, LI 4, LI 10, SP-6, LR-3, ST-36, GB-26, CP-15, ST-28 and Ren-4 points were stimulated using sterile, disposable, steel needles with a single-sided point and length and diameter of 0.30x40 mm and 0.25x25 mm, placed bilaterally in a single session at a depth of 10-50 mm and angled at 45-90˚ to the skin. ACU was applied to these points for 20 minutes using Acutens SMS-205, 5-channel, 1-200 Hz, 1-10 mA, at 1-20 Hz frequency and current at maximum 10 mA intensity^(16)^. The HSG procedure was applied within 3 hours of the ACU application.

For the patients in the IMDS group, 75 mg diclofenac sodium (Diclomec®, Abdi İbrahim Medicin, Turkey) was administered intramuscularly one hour before the HSG procedure^(17)^.

No medication or procedure was applied to the control group patients before HSG.

The randomisation of patients to the 3 groups was performed by a single physician (M.G.) blinded to the patients. HSG was applied in the Outpatients clinic at 1-3 days after the end of menstruation, in the follicular phase of the menstrual cycle. HSG was performed under fluoroscopy guidance with the patient in the lithotomy position. A sterile speculum was placed in the vagina, and following observation of the cervix, local cleaning was applied with 3% povidone-iodine. Then, depending on the cervix position, the anterior or posterior cervical lip was held with a tenaculum, and a Rubin HSG cannula was gently placed in the cervical canal. The speculum was removed and 10-20 mL water-soluble, radio-opaque solution (Urografin® 76% 100 mL, Bayer AG, Turkey) was used as a contrast medium and injected slowly under spot fluoroscopy. After completion of the procedure, all the instruments were removed and the patient was transferred to a bed.

A Visual Analog scale (VAS) was used in the evaluation of pain severity, and the State-trait Anxiety inventory- state (STAI-S) in the evaluation of patient anxiety. All the VAS and STAI-S scoring was performed by a single physician (A.U.) blinded to the groups. In the VAS system, the patient is instructed to place a mark on a 100 mm horizontal line corresponding to the level of pain felt, where 0 =no pain and 10 =intolerable pain. The questions on the STAI-S form were asked directly to the patients and the results were recorded. Both evaluations were applied at 1 min and 5 min before the HSG procedure and at 30 minutes after the procedure and all the results were recorded. In the evaluation of anxiety, the changes over the specified time period were evaluated.

### Statistical Analysis

Data obtained in the study were analysed statistically using SPSS 22.0 vn 22 software (IBM Statistics for Windows version 22, IBM Corporation, Armonk, NY, USA) and PAST 3 software (Hammer, *Ø*., Harper, D.A.T., Ryan, P.D. 2001. Paleontological statistics). To assess the conformity of data to normal distribution, the Shapiro-Wilk test was applied to data with single variables and the Mardia test (Dornikand Hansen omnibus) to data with multiple variables. The Levene test was used to evaluate variance homogeneity. Parametric methods were used in the analyses of variables with homogenous variance and normal distribution and non-parametric methods were used for variables not showing homogenous variance and normal distribution. In the comparisons of independent multiple groups with each other, the One-Way ANOVA (Robust test: Brown-Forsythe) and Kruskal-Wallis tests were used, and for the post hoc analyses, the non-parametric post hoc test (Miller, 1966). To examine the interaction of repeated measurements of dependent variables according to the groups, the General Linear Model Repeated Anova (Wilks Lambda) was used and the LSD test for post hoc analysis. Quantitative data were presented in the tables as mean ± standard deviation and median range (IQR or minimum-maximum) values, and categorical data as number (n) and percentage (%). The data were examined at a 95% confidence interval. A value of p<0.05 was accepted as statistically significant.

## Results

**Figure 1:**
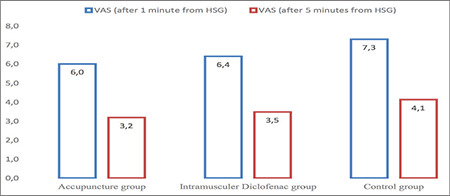
VAS scores 1 minute before and 5 minutes after HSG VAS: Visual Analog scale, HSG: Hysterosalpingography

**Figure 2:**
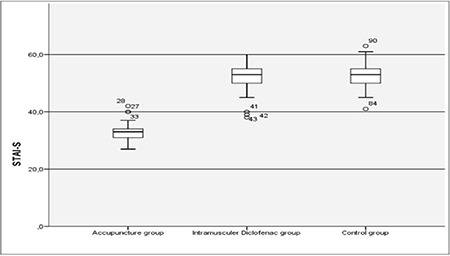
STAI-S scores 5 minutes after HSG HSG: Hysterosalpingography, STAI-S: State-trait Anxiety inventory -state

As shown in Table 1, there was no statistical difference found among the mean age, infertility duration, gravida and smoking status of the patients participating in the study (all p>0.05). When the VAS scores 1 and 5 minutes after the procedure were examined, it was found that the values ​​in the control group were statistically significantly higher than the ACU and IMDS groups, and there was no difference between the ACU and IMDS groups (p<0.001 and p=0.002, respectively). There was no statistical difference found between the VAS scores at the 30^th^ minute after the procedure (p=0.625) (Table 2, Figure 1). There was no statistically significant difference found between the STAI-S values ​​determined at the first hour of the procedure, that is, just before the application of ACU and intramuscular Diclofenac (p=0.563)**. **When STAI-S values ​​determined at the 5^th^ minute before the procedure, that is, after ACU in the ACU group, and after Diclofenac injection in the IMDS group, were examined the values ​​in the ACU group were found to be statistically significantly lower than the IMDS and control groups while that difference was not determined between the IMDS and control groups (p<0.001) (Figure 2). STAI-S scores 5 minutes before the procedure decreased only in the ACU group compared to the STAI-S score 1 hour before the procedure, but increased in the IMDS and control groups. When the STAI-S scores 30 minutes after the HSG procedure were examined, the values ​​in the ACU group were found to be statistically significantly lower than the IMDS and control group, similar to the 5^th^ minute before the procedure, but this difference was not determined between the IMDS and control groups (p<0.001) (Table 3). When the changes in the STAI-S scores (between 1 hour before-5 minutes ago, 1 hour before-30 minutes later and 5 minutes before-30 minutes later, respectively) were analyzed, the changes in STAI-S scores in the ACU group were found to be statistically significant for all three change values ​​than the IMDS and control group while this difference was not determined between the IMDS and control groups (p<0.001, p<0.001 ve p<0.001, respectively) (Table 3).

## Discussion

The results of the study showed that ACU reduced the pain scores before and after the HSG procedure as effectively as the NSAID diclofenac. It was also determined that unlike diclofenac, ACU had the effect of reducing anxiety related to the procedure.

There are many studies on the subject of pain relief in HSG that have investigated the types of pharmacological drugs used and the methods of use. A systematic Cochrane review in 2015 on the subject of pain releief in HSG analysed 23 prospective, randomised studies. Although the level of evidence was low, it was concluded that topical anaesthetics and intravenous opioids could be effective in reducing pain during the procedure, but this effect was not seen after the procedure. It was reported that there was not sufficient evidence that other analgesic methods could be effective^(10)^. In a review by Ahmad et al.^(9)^ on the subject of pain-relief in office-setting gynaecological interventions, there was reported to be no benefit of oral NSAIDs within the first 30 mins and 30 mins after the procedure, but when local anaesthetics were used, it was shown that even if not in the first 30 mins, there could be beneficial effects after 30 mins.

The majority of studies in literature related to reducing pain with non-pharmacological methods are studies where catheters have been used. It has been suggested that catheters used during HSG could have different effects on pain. In studies by Austin et al.^(18)^ and Varpula^(19)^, it was determined that the use of flexible balloon catheters instead of a traditional metal cannula was not effective in reducing pain, while De Mello et al.^(20)^ reported in a study published in 2006 that the use of flexible balloon catheters resulted in less pain. Stoop et al.^(21)^ investigated the efficacy of fast-release orodispersible tramadol, as a different analgesic method, in cases where traditional metal cannula and balloon catheter were used, and reported that it was effective independently of the type of catheter used.

ACU is a complementary medicine application with increasingly widespread use. There have started to be wide areas of use especially in the elimination of pain symptoms. Previous studies have shown that the effect mechanisms of ACU are formed on a biological basis. Cheng and Pomeranz^(22) ^reported that the analgesic efficacy of ACU occurred by increasing endogenous opioids and demonstrated that this effect could be removed with naloxane, which is an opioid antagonist. Using functional magnetic imaging technology, another study showed that the stimulation of ACU points affected the the limbic system and structures in both the cortical and subcortical areas in the brain^(23)^. In a clinical study, Cho et al.^(24)^ determined activity signals in the cingulate gyrus and thalamic region with pain stimuli, and following ACU, reported a decrease both in the signals and in the pain felt by the patient.

Although there are no studies in literature examining the effect of ACU on anxiety engendered by the application of HSG, there are studies related to the effect of lowering anxiety in general. The hypotheses of some of these studies are explained by biochemical mechanisms and some by physiological parameters. Yuan et al.^(25)^ investigated changes in plasma adrenocorticotropic hormone, corticosteroid and platelet 5-HT levels in response to anxiety treatment. Comparisons were made of ACU, pharmacological treatment and combined treatment groups, and similar results were found in the ACU group and the pharmacological treatment group. That the side-effects seen in the pharmacological treatment group were not seen in the ACU group was emphasised as a positive aspect. The effects of ACU on anxiety were investigated by observing changes in the parameters of oxygen saturation and heart rate by Karst et al.^(26)^ and in heart rate and skin conductivity by Shayestehfar et al.^(27)^. As ACU reduced heart rate in both groups, it was concluded to be effective on anxiety.

To the best of our knowledge, this is the first study in literature to have investigated ACU on this subject. However, there were some limitations to the study, the first of which is that the different stages of HSG (speculum placement, tenaculum placement, opaque medium administration) were not evaluated with VAS. In addition, the side-effects of NSAIDs and ACU were not examined, and when examining the pain scores, the HSG results were not taken into consideration.

## Conclusion

Although the use of ACU is restricted in most clinics because of the need for trained practitioners, equipment and time, as it has a similar effect to other analgesics in reducing pain, and effectively reduces anxiety, it can be used in HSG in suitable clinics.

## Figures and Tables

**Table 1:**
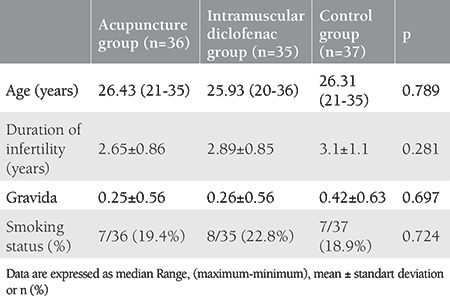
Demographic characteristics of the cases

**Table 2:**
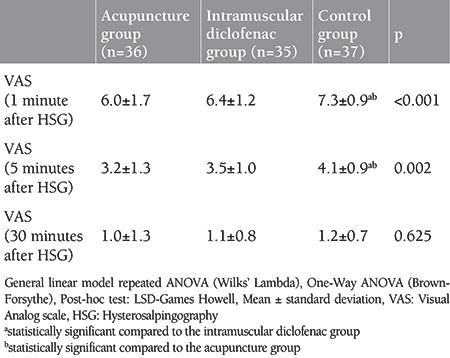
VAS scores at 1, 5, and 30 mins after HSG

**Table 3:**
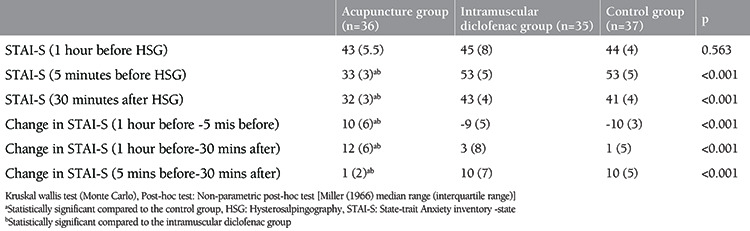
STAI-S values at 1 hour and 5 minutes before and 30 minutes after HSG and the changes in those intervals
